# DNA Vaccines Expressing the Envelope and Membrane Proteins Provide Partial Protection Against SARS-CoV-2 in Mice

**DOI:** 10.3389/fimmu.2022.827605

**Published:** 2022-02-24

**Authors:** Jinni Chen, Yao Deng, Baoying Huang, Di Han, Wen Wang, Mengjing Huang, Chengcheng Zhai, Zhimin Zhao, Ren Yang, Ying Zhao, Wenling Wang, Desheng Zhai, Wenjie Tan

**Affiliations:** ^1^School of Public Health, Xinxiang Medical University, Xinxiang, China; ^2^National Health Commission (NHC) Key Laboratory of Medical Virology, National Institute for Viral Disease Control and Prevention, China CDC, Beijing, China; ^3^Basic Medical College, Inner Mongolia Medical University, Hohhot, China; ^4^School of Public Health, Baotou Medical College, Baotou, China; ^5^School of Pharmacy, Xinxiang Medical University, Xinxiang, China

**Keywords:** SARS-CoV-2, DNA vaccine, envelope protein, membrane protein, humoral response, cellular response

## Abstract

The coronavirus disease 2019 (COVID-19) pandemic caused by the severe acute respiratory syndrome coronavirus 2 (SARS-CoV-2) has become a public health emergency of international concern, and an effective vaccine is urgently needed to control the pandemic. Envelope (E) and membrane (M) proteins are highly conserved structural proteins among SARS-CoV-2 and SARS-CoV and have been proposed as potential targets for the development of cross-protective vaccines. Here, synthetic DNA vaccines encoding SARS-CoV-2 E/M proteins (called p-SARS-CoV-2-E/M) were developed, and mice were immunised with three doses *via* intramuscular injection and electroporation. Significant cellular immune responses were elicited, whereas no robust humoral immunity was detected. In addition, novel H-2d-restricted T-cell epitopes were identified. Notably, although no drop in lung tissue virus titre was detected in DNA-vaccinated mice post-challenge with SARS-CoV-2, immunisation with either p-SARS-CoV-2-E or p-SARS-CoV-2-M provided minor protection and co-immunisation with p-SARS-CoV-2-E+M increased protection. Therefore, E/M proteins should be considered as vaccine candidates as they may be valuable in the optimisation of vaccination strategies against COVID-19.

## Introduction

Severe acute respiratory syndrome coronavirus 2 (SARS-CoV-2), the cause of coronavirus disease 2019 (COVID-19), is the third novel betacoronavirus belonging to highly pathogenic human coronaviruses that have caused public health crises in the past 20 years (1). It transmits more efficiently among the population compared with its predecessors, SARS-CoV and Middle East respiratory syndrome coronavirus. Globally, SARS-CoV-2 has infected more than 260 million people, resulting in more than 5 million deaths worldwide as of November 2021 (2). Therefore, the rapid development of effective therapies and vaccines against SARS-CoV-2 and emerging coronaviruses is a critical global priority (3).

Human coronaviruses are enveloped positive-sense RNA viruses. The genetic components of coronaviruses encode four major structural proteins—spike (S), nucleocapsid (N), envelope (E), and membrane (M) proteins. The S protein is the target of neutralising antibodies during infection and is important for the development of coronavirus vaccines (4). However, SARS-CoV-2 variants are emerging in different parts of the world, posing a new threat of increased virus spread and the potential to escape vaccine-induced immunity. Most of the mutations in these variants are within the S protein (5); thus, raises the concern that monovalent vaccines targeting only the S protein may not be the most optimal strategy for conferring protection against continually emerging variants (6).

The E protein is a small ion channel-forming membrane protein (75 amino acids; ∼8.4 kDa) that plays a significant role in viral morphogenesis and assembly (6). The M protein is the most abundant protein and is approximately 222 amino acid residues in length. It interacts with other structural viral proteins and plays a central organising role in coronavirus assembly (7). Both proteins are highly conserved structural proteins of SARS-CoV-2 and SARS-CoV (8). Several reports have shown that the co-synthesis of E and M proteins is sufficient for virus-like particle assembly (9–11). Therefore, the E/M proteins are potential targets for the development of SARS-CoV-2 cross-protective vaccines. However, few studies have considered these proteins as major targets for the development of SARS-CoV-2 vaccines (12).

A variety of SARS-CoV-2 vaccines are currently being developed and some have been approved, including inactivated-, subunit-, vector-, mRNA-, and DNA vaccines (13, 14). Although several vaccines against COVID-19 are promising, developing more effective and cross-protective vaccines is urgently required. Synthetic DNA vaccines are developed at an accelerated rate because of the quick design of multiple candidates for preclinical testing in comparison to other vaccines (15–17). Currently, to the best of our knowledge, the immunogenicity and protective potential of synthetic DNA vaccines encoding the SARS-CoV-2 E/M proteins have not been reported.

To explore the immune protective potential of the SARS-CoV-2 E and M proteins as vaccine targets synthetic DNA vaccines expressing the E and M proteins were developed and their immunogenicity and protective efficacy in mice were evaluated in this study.

## Materials and Methods

### Construction of DNA-Based COVID-19 Vaccines

**Figure 1:**
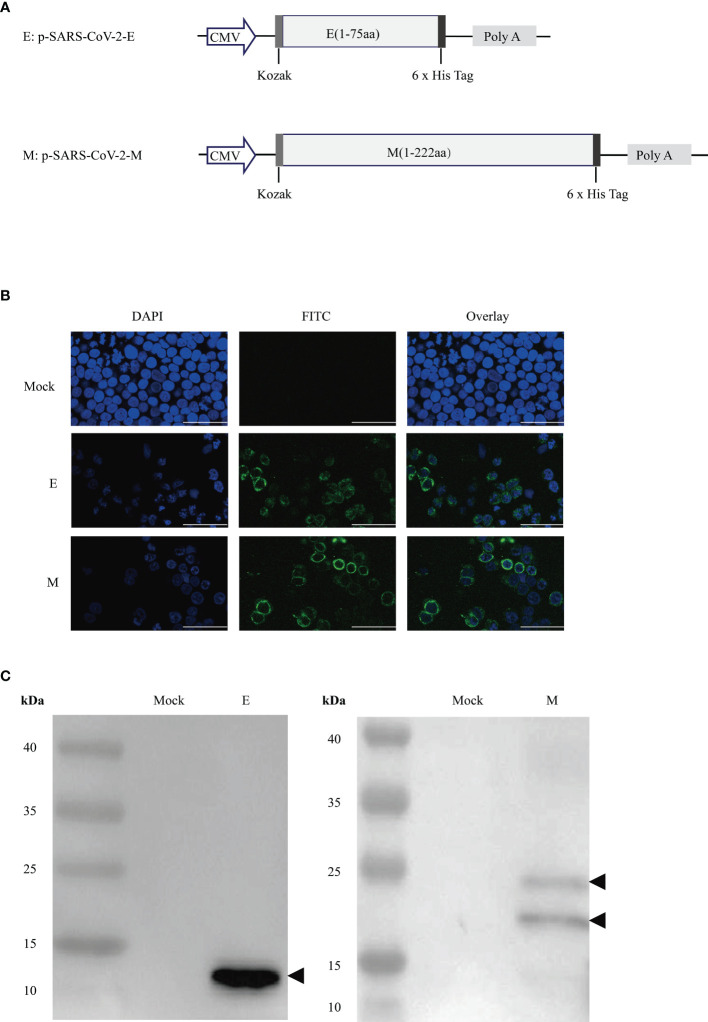
Design and expression of recombinant DNA-based SARS-CoV-2 E/M proteins vaccine constructs. **(A)** Schematic diagram of the recombinant DNA-based vaccines encoding severe acute respiratory syndrome coronavirus 2 (SARS-CoV-2) envelope (E)/membrane (M) protein genes. **(B)** E/M protein expression in DNA vaccines were tested by indirect immunofluorescence staining and **(C)** western blot in 293T cells transfected with either of the pSARS-CoV-2-E/M plasmids.

The full-length genes encoding the SARS-CoV-2 E and M proteins (GISAID, No. EPI_ISL_402119) were synthesised using a mammalian-optimised codon with a N-terminal Kozak sequence(GCCACC) followed by initiation codon(ATG) and a C-terminal 6x His tag (GenScript Co., Nanjing, China). Subsequently, they were inserted into the eukaryotic expression vector, pcDNA3.1 (+), *via Hin*dIII and *Xba*I digestion and named as p-SARS-CoV-2-E or p-SARS-CoV-2-M (**Figure 1A**). All DNA vaccine sequences were confirmed by Sanger DNA sequencing, and vaccines were expanded using endotoxin-free Maxiprep kits (Qiagen, Beijing, China). The expression of the E/M proteins was identified by an indirect immunofluorescence assay (IFA) and western blotting.

### Indirect IFA

Human embryonic kidney 293T cells were grown in Dulbecco’s modified Eagle’s medium (Hyclone, South Logan, UT, USA) containing 10% foetal bovine serum (Gibco, NY, USA) and 1% penicillin-streptomycin (Gibco, Grand Island, NY, USA) in a 5% CO_2_ incubator at 37°C. 293T cells were transfected with either the p-SARS-CoV-2 E/M or pcDNA3.1 (empty/mock) vector using jet PRIME transfection reagent (Polyplus, Illkirch, France). Cells were fixed in pre-cooled 4% paraformaldehyde, mobilised in 0.2% Triton X-100, and blocked by 10% goat serum in phosphate-buffered saline. Anti-6X His tag antibody (Abcam, Cambridge, UK) diluted at 1:50 was used as the primary antibody. After incubation, cells were washed and incubated with secondary antibodies [fluorescein isothiocyanate(FITC)-labelled goat anti-rabbit IgG] and 0.1% 4′,6-diamino-2-phenylindol (DAPI) at 37°C for 10 min. Fluorescent images were acquired using a Leica TCS SP8 confocal microscope with LAS software (Leica Biosystems, Wetzlar, Germany).

### Western Blot

The expression of the E or M protein was confirmed by western blotting, as previously described (18). The anti-6× His tag antibody (Abcam, UK) diluted at 1:500 or serum antibodies (diluted at 1/10) from DNA-immunised mice were used as primary antibodies. A 1:5,000 dilution of anti-mouse horseradish peroxidase-conjugated antibody (Sigma Aldrich, St Louis, MO, USA) was used as the secondary antibody. The membranes were developed with a chemiluminescent substrate and analysed using a chemiluminescent imager.

### Immunisations and Challenge

All experiments were approved by the Committee on the Ethics of Animal Experiments of the Chinese Centre for Disease Control and Prevention where all live SARS-CoV-2 mice experiments were performed in animal biosafety level 3 containment laboratories at the National Institute for Viral Disease Control and Prevention.

For animal immunisation, 6-week-old female BALB/c mice (SPF grade) were purchased from the Beijing Vital River Laboratory Animal Technology, housed, and vaccinated at 25°C in a light-cycled facility (12 h light/12 h dark).”

**Figure 2:**
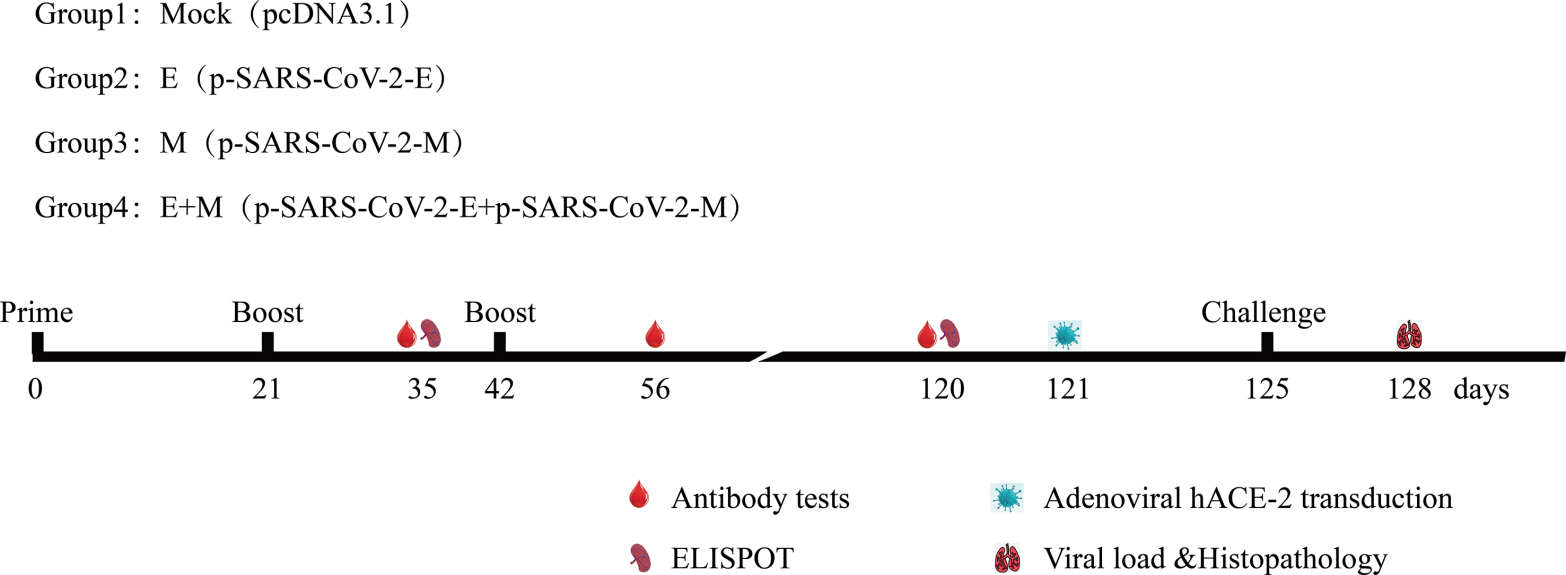
Immunisation and challenging schema of recombinant DNA-based SARS-CoV-2 E/M proteins coronavirus disease 2019 vaccines. Vaccination, challenging, and blood/tissue sampling time course. BALB/c mice were divided randomly into groups.

Mice were divided randomly into groups (**Figure 2**) and immunised with pcDNA3.1 (+), p-SARS-CoV-2-E/M alone or co-immunised with p-SARS-CoV-2-E+M, on days 0, 21, and 42 *via* intramuscular injection plus electroporation (35μg/50μl) (19, 20). In brief, DNA vaccine were injected into the TA muscle of mice and were immediately pulsed with electricity using a two-needle array electrode (ECM830; BTX) with needles that were 5 mm apart. Their spleens were processed to measure cellular immune responses to E or M antigens, and their sera were collected and used to analyse humoral immune responses.

Viral challenge experiments were conducted as described previously (21). Ad5-hACE2-transduced SARS-CoV-2 mice were intranasally infected with 1×10^5^ median tissue culture infective dose (TCID50) of SARS-CoV-2 (Wuhan/IVDC-HB-02/2019) in a total volume of 50 μL.

### Interferon Gamma (IFN-γ) Enzyme-Linked Immune Absorbent Spot (ELISpot) Assay

Either the E peptide pool or M peptide pool spanned the entire protein as consecutive 15-mers overlapping by 10 amino acids were synthesised by Scilight Biotechnology LLC. Each purified peptide of the peptide pool was at 2.5 mg per vial. The peptides were dissolved in dimethyl sulfoxide (DMSO) at a concentration of 50 mg/ml and stored at -80°C. The experiment was conducted as described previously (22).

### Enzyme-Linked Immunosorbent Assay (ELISA)

Synthetic extracellular peptides of the E/M proteins coupled with bovine serum albumin (synthetized by Scilight Biotechnology LLC) or E/M proteins (purchased from Unique Biotechnology LLC) diluted in carbonate buffer (0.1 M; pH 9.6) were used to coat 96-well enzyme immunoassay/radioimmunoassay plates (Thermo Fisher Scientific, Waltham, MA, USA) overnight at 4°C. ELISA was conducted as described previously (21).

### SARS-CoV-2 Neutralisation Assay

The experiment was conducted in a biosafety level 3 laboratory as previously described (21).

### Evaluation of Protection in Mice Post SARS-CoV-2 Challenge

Three days post-challenge, mice were euthanised, and necropsy was performed. Lungs of mice were harvested after sacrifice (four mice per group). Partial tissues were used for nucleic acid extraction and real-time fluorescence RT-PCR to quantify the relative amount of viral RNA in lungs as previously described (21). The TCID50 of the virus in samples was determined as previously described (21). Remaining tissue samples were fixed in a 4% formalin solution and sent to the College of Veterinary Medicine, China Agricultural University, for the preparation of haematoxylin and eosin-stained sections (four mice per group) for pathological evaluation indicated by the International Harmonisation of Nomenclature and Diagnostic Criteria (INHAND) scores.

### Statistical Analysis

All statistical analyses were performed using GraphPad Prism 7.0 (GraphPad Prism Software Inc., San Diego, CA, USA). One-way ANOVA with Dunnett’s multiple comparisons test was performed to evaluate the statistical significance of differences among groups. Statistical significance was set at *P*<0.05.

## Results

### Characterisation of DNA Vaccines

Expression of E and M proteins in 293T cells transfected with p-SARS-CoV-2-E/M was detected by IFA (**Figure 1B**) and western blotting **(****Figure 1C**). IFA showed the expression of E and M proteins in the membrane and endoplasmic area of HEK-293T cells transfected with p-SARS-CoV-2-E/M. Western blot results revealed approximately 10 kDa and 25 kDa bands that were predicted as E and M proteins based on the molecular weight in the lysates of HEK-293T cells transfected with p-SARS-CoV-2-E/M. Two bands of M proteins means that some M proteins can undergo maturation leading to N-glycosylated M proteins found in infected cells (23). This has also been observed in the case of SARS-CoV-2, revealed by two specific bands for M proteins using immunoblotting (24). No protein expression was detected in pcDNA3.1 (+)-transfected cells.

### Significant and Sustained E-/M-Specific T-Cell Responses Induced by DNA Vaccination

**Figure 3:**
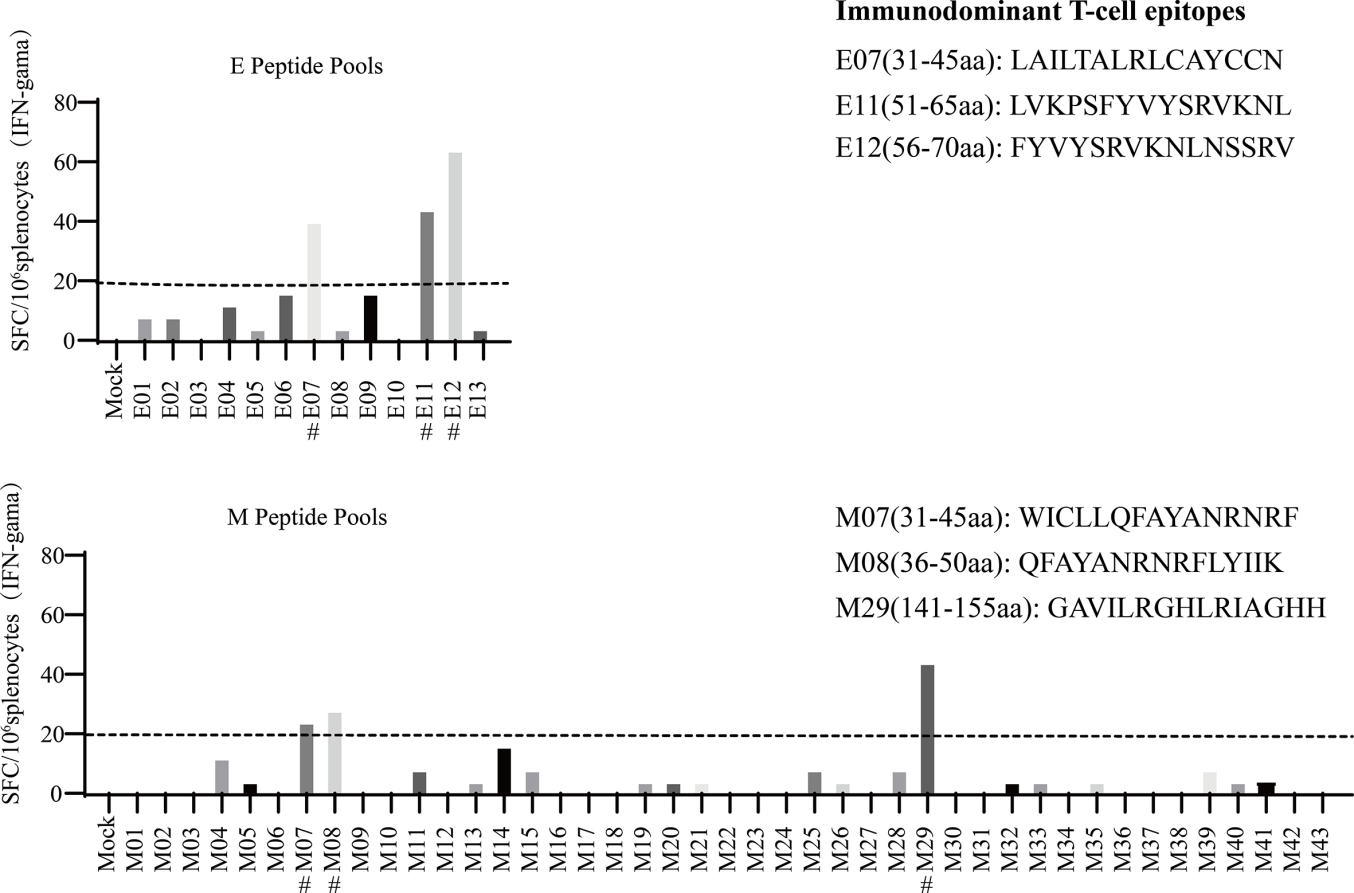
Mapping severe acute respiratory syndrome coronavirus 2 (SARS-CoV-2) T-cell epitopes in BALB/c mice. The fifty-six 15-mer overlapping peptides that cover the entire sequence of the SARS-CoV-2 E/M proteins were used in an enzyme-linked immune absorbent spot (ELISPOT) assay to measure the immunodominant T-cell epitopes. Candidate T-cell epitopes are labelled with #.

To systemically analyse the H-2d-restricted T-cell epitopes for SARS-CoV-2 E and M proteins, peptide pools consisting of 10 consecutive 15-mer overlapping peptides of the E/M proteins were used for IFN-γ ELISpot assay screening in BALB/c mice after vaccination with p-SARS-CoV-2-E/M. Individual peptide reactivity analysis showed that E-07, E-11, E-12, M-07, M-08, and M-29 contained immunodominant epitopes, the amino acid sequences of which are shown in **Figure 3**.

**Figure 4:**
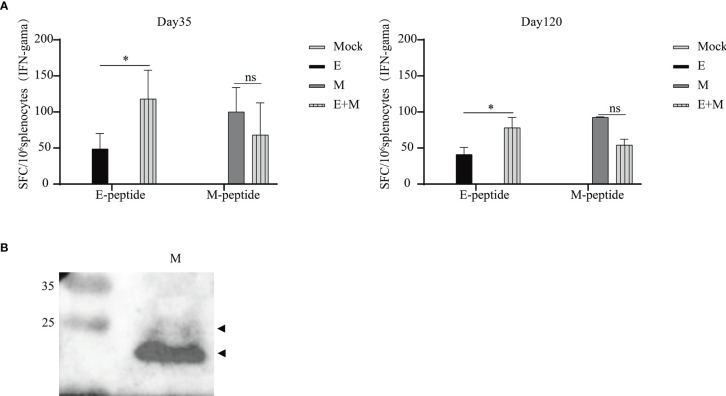
T and B cell immunity induced upon immunisation of mice with severe acute respiratory syndrome coronavirus 2 (SARS-CoV-2) envelope (E) and/or membrane (M) proteins. **(A)** Splenocytes were isolated from mice (n=3 or 4 mice per group per time point) and stimulated with E/M peptides at days 35 and 120 after the 1st vaccination. **(B)** M protein-specific antibodies were detected by western blot analysis of purified SARS-CoV-2 particles (inactivated vaccine stock). The statistical analysis among groups was analysed by two-way ANOVA after Tukey’s multiple comparison (*P<0.05). ns, no significance.

As shown in **Figure 4A**, a significant level of IFN-γ production was observed in the p-SARS-CoV-2-E/M-immunised and p-SARS-CoV-2-E+M-immunised groups, while no IFN-γ secretion was detected in the mock group. Co-immunisation with p-SARS-CoV-2-E+M induced higher levels of E protein-specific IFN-γ secretion than immunisation with p-SARS-CoV-2-E alone on days 35 (E+M: 118 spot-forming units [SFU]/10^6^ splenocytes vs. E: 49 SFU/10^6^ splenocytes; *P*<0.05) and 120 (E+M: 78 SFU/10^6^ splenocytes vs. E: 41 SFU/10^6^ splenocytes; *P*<0.05). However, co-immunisation with p-SARS-CoV-2 E+M did not induce higher levels of M-protein-specific IFN-γ secretion than immunisation with p-SARS-CoV-2 M alone on days 35 (E+M: 68 SFU/10^6^ splenocytes vs. M: 100 SFU/10^6^ splenocytes) and 120 (E+M: 54 SFU/10^6^ splenocytes vs M: 93 SFU/10^6^ splenocytes).

### IgG and Neutralizing Antibody Responses Induced by DNA Vaccination

ELISA results from plates coated with E/M proteins (AtaGenix Laboratories, Wuhan, China) or E/M peptides (Scilight Biotechnology LLC,Beijing, China) did not indicate robust E/M protein-specific antibody responses after the first or second vaccination (data not shown). Moreover, neutralising anti-E/M IgG were not detected in the sera (1:10 dilution) of immunised mice (data not shown). However, antibodies against M protein in mice could be detected by western blot (**Figure 4B**), and the M-specific band (as predicted by size) was observed when purified SARS-CoV-2 particles (inactivated vaccine stock) were loaded and incubated with serum (diluted at 1/10) of p-SARS-CoV-2-M-immunised mice.

### Co-Immunization With p-SARS-CoV-2-E+ M Induced Partial Protection After Challenge

**Figure 5:**
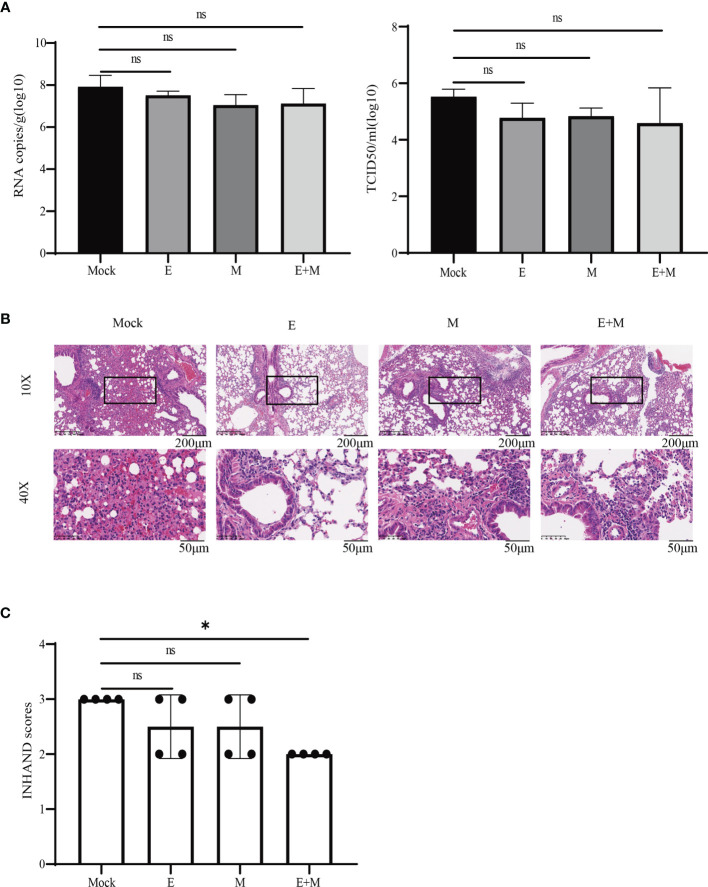
Immunisation protects mice from live severe acute respiratory syndrome coronavirus 2 (SARS-CoV-2) challenge. **(A)** Tissue viral loads and **(B)** histopathology analysis of SARS-CoV-2-challenged mice. **(C)** International Harmonisation of Nomenclature and Diagnostic Criteria (INHAND) scores of challenged mice organs, on a severity scale of 0–3 (none, mild, moderate, and severe). Statistical significance for groups of a one-way ANOVA after Dunnett’s multiple comparison correction is shown (*P < 0.05). ns, no significance.

We determined whether there was enhanced protection against SARS-CoV-2 challenge in p-SARS-CoV-2-E/M- and p-SARS-CoV-2-E+M-immunised mice, compared to the control group by evaluating protective effects of the DNA vaccine in mice on day 125. Tissue viral load (RNA copies and TCID50) and histopathological changes were evaluated (**Figure 5**). Lung viral load in DNA vaccine-immunised mice did not significantly decrease compared to that in the control group (**Figure 5A**). Lung histopathology demonstrated that mice in the control group had ruptured pulmonary alveoli, excessive mucus production, and immune cell infiltration. In contrast, DNA vaccine-immunized mice had milder histopathological changes (**Figure 5B**). INHAND scores of all DNA vaccine-immunised groups were lower than those of the control group. Notably, mice co-immunised with p-SARS-CoV-2-E+M exhibited the mildest histopathological changes and lowest INHAND scores compared to mice immunised with p-SARS-CoV-2-E/M alone. Moreover, the INHAND scores of the SARS-CoV-2-E+M vaccine group were significantly lower than those of the control group (***P***<0.05; **Figure 5C**). Taken together, both p-SARS-CoV-2-E and p-SARS-CoV-2-M immunisation provided partial protection in mice after SARS-CoV-2 challenge, and co-immunisation with p-SARS-CoV-2 E+M enhanced this protection.

## Discussion

In this study, two DNA vaccines expressing the SARS-CoV-2 E and M proteins were developed. The data showed that considerable cellular immune responses were elicited, whereas no robust humoral immunity was detected in BALB/c mice. In addition, six novel H-2d-restricted T-cell epitopes were identified in E and M proteins. Co-immunisation with two DNA vaccines expressing E and M proteins provided partial protection against SARS-CoV-2. To the best of our knowledge, this is the first study to evaluate the immune protective potential of the SARS-CoV-2 E and M proteins as vaccine targets.

Previous studies have suggested that SARS-CoV-2-specific T-cells play a key role in COVID-19 resolution and modulation of disease severity (25, 26). The definition of SARS-CoV-2-specific T-cell epitopes is important for evaluating the potential influences of mutations on acquired immunity and vaccine efficacy. The immunodominant T-cell epitopes in the E and M antigen regions have only been determined in a few studies (27, 28). M protein-specific cellular immune responses have previously been reported from SARS−CoV vaccination in mice (29, 30), but there are no reports on similar responses to the E protein. Previous studies have reported that overlapping peptide pools of the E and M proteins induce SARS-CoV-2-reactive T-cell responses in humans with COVID-19 (25, 31). Immunoinformatic analyses in humans have identified SARS-CoV-2 E-specific (LVKPSFYVYSRVKNL/FYVYSRVKNLNSSRV/FLLVTLAILTALRLC) and M-specific (RGHLRIAGHHLGRCD) T-cell epitopes. These peptides elicited T-cell responses in 33%, 36%, 22%, and 72% of patients with COVID-19 and overlapped with E11, E12, E07, and M29, respectively (25, 27, 28, 32–34). Another predicted M protein-specific T-cell epitope (LLQFAYANRNRFLYI) overlapped with M07 and M08 (27, 28), which were identified in this study. However, a few predicted E/M protein-specific T-cell epitopes were not confirmed in this study, possibly due to the differences between mice and humans. Notably, one study with results inconsistent with our data reported that SARS-CoV-2 E/M-specific peptides were not able to stimulate CD4^+^ and CD8^+^ T-cells from virus replicon particle-vaccinated BALB/c mice (35). This may reflect differences in target protein expression between DNA- and virus replicon particle-based vaccines. The multiple amino-acids sequence alignment have been performed for immuno-dominant epitopes for E and M identified in our study, which shows a conservation of E07 (100% identity) between SARS-CoV and SARS-CoV-2. However, the suitability of E11, E12, M07, M08, and M29 for SARS-CoV requires further testing (36). Variant of concern (VOC) is a variant for which there is evidence of an increase in transmissibility and disease severity, significant reduction in neutralization by antibodies generated during previous infection or vaccination (37). For E protein among 5 of SARS-CoV-2 VOCs, there is only one substitution (T9I) in the omicron variant, one substitution (P71L) in the Beta variant. For M protein, there are three substitutions (D3G, Q19E, and A63T) in the omicron variant, one substitution (I82T) in the Delta variant (37, 38). Fortunately, most immuno-dominant epitopes for E and M identified in our study are conserved between SARS-CoV and VOCs of SARS-CoV-2. Notably, persistence of T-cell responses against E or M was shown by Day 120 data until weeks 11 after vaccination (**Figure 4**).

A few studies have tested the potential of the E protein as a vaccine target. One SARS−CoV vector vaccine study reported that none of the animals immunised with vaccines expressing E, ME, or SME proteins induced antibody responses specific to the E protein (39). Therefore, we speculated that the SARS-CoV-2 E protein has a limited ability to induce a humoral immune response. A few studies have reported that SARS-CoV M protein-based vaccines can induce antibody responses in immunised animals (29, 30, 39, 40). Of note, neutralising antibody titres specific to SARS-CoV M protein were detected in immunised animals and patients with SARS (41, 42). Previous immunoinformatic studies identified M-specific B-cell epitopes, which were confirmed by ELISA using convalescent sera from patients who previously had COVID−19 (28). However, there were no studies that identified E-specific B-cell epitopes. One study identified two linear E-specific B-cell epitopes (CoV2_E-1 and CoV2_E-1.1) by immunoinformatic prediction, but statistical analysis revealed that antibody responses against these epitopes in convalescent sera were not significantly higher than those in healthy control sera (43). Our study revealed that no significant IgG or neutralizing antibody against E/M were detected in vaccinated mice. This may be due to limited B-cell epitopes relatively smaller molecular weight of E protein, and weaker immunogenicity of DNA vaccination.

One study communicated that immunisation with bovine-human parainfluenza virus type 3 expressing the S protein provided complete and partial protection against SARS-CoV in the lower and upper respiratory tract, respectively. This was augmented slightly by co-expression with M and E. However, the expression of M, E, or M plus E in the absence of S did not confer detectable protection against SARS-CoV (39). Notably, hamsters immunised with a vaccine co-expressing the M and N proteins were protected against severe weight loss and lung pathology and had reduced viral loads in the oropharynx and lungs after SARS-CoV-2 challenge (12). Our study demonstrated that p-SARS-CoV-2-E or p-SARS-CoV-2-M immunisation provided minor protection (indicated by mild lung tissue pathology), and co-immunisation with p-SARS-CoV-2-E+M exhibited even more protection (indicated by the mildest histopathological changes and lowest INHAND scores), although no drop in lung tissue virus titre was detected in DNA-vaccinated mice after challenge with SARS-CoV-2. Furthermore, the longevity of protective immunity provided by DNA vaccines expressing SARS-CoV-2 E/M was supported here, even though the challenge study was carried out nearly 3 months post vaccination.

This study had limitations. We only observed the DNA vaccine strategy in BALB/c mice, and because of the low immunogenicity of DNA vaccines, the protection efficacy could be further explored in subunit vaccines, vector vaccines, or novel combinations of DNA and other vaccines, and future studies should estimate the immunity effect in other animal models. Importantly, additional research is needed to understand the molecular mechanisms of the E/M-mediated immune protective effect after SARS-CoV-2 challenge so as to harness this knowledge to optimise COVID-19 vaccine design.

In summary, we present a detailed immunological study of the SARS-CoV-2-specific immune response against E and M proteins after DNA vaccination. This is the first experimental report to support immune protection against SARS-CoV-2 provided by the specific cellular immune response against E and M proteins in the absence of an obvious humoral immune response. The emergence of SARS-CoV-2 variants has raised concerns about the potential loss of protection from COVID-19 vaccines targeting only the highly mutated S protein. The role of conserved structural proteins of SARS-CoV-2, including E/M protein, is worthy of attention in vaccine design and application since vaccine-induced T- cell responses against conserved epitopes will be unaffected by SARS-CoV-2 variants (39). Our results will lay a strong foundation for the development of a cross-protective COVID-19 vaccine for controlling current and emerging variants of concern, as well as for preventing future β-coronavirus pandemics.

## Data Availability Statement

The original contributions presented in the study are included in the article/supplementary material. Further inquiries can be directed to the corresponding authors.

## Ethics Statement

The animal study was reviewed and approved by Committee on the Ethics of Animal Experiments of the Chinese Center for Disease Control and Prevention.

## Author Contributions

WT, DZ, and YD conceived of the study. JC, DH, WW, MH, CZ, ZZ, RY, YZ, and BH performed the experiment. JC, WW and BH analysed the data. JC drafted the manuscript. DZ and WT revised the manuscript. All authors reviewed and approved the final manuscript.

## Funding

This work was supported by grants from the National Natural Science Foundation of China (82041041, 82061138008). Partly supported by the Support Project of Scientific and Technological Innovation Team in Universities of Henan Province [No. 20IRTSTHN027].

## Conflict of Interest

The authors declare that the research was conducted in the absence of any commercial or financial relationships that could be construed as a potential conflict of interest.

## Publisher’s Note

All claims expressed in this article are solely those of the authors and do not necessarily represent those of their affiliated organizations, or those of the publisher, the editors and the reviewers. Any product that may be evaluated in this article, or claim that may be made by its manufacturer, is not guaranteed or endorsed by the publisher.

## Abbreviations

COVID-19, coronavirus disease 2019; E, envelope; ELISA, enzyme-linked immunosorbent assay; IFA, immunofluorescence assay; IFN-γ, interferon gamma; INHAND, International Harmonisation of Nomenclature and Diagnostic; M, membrane; SARS-CoV-2, severe acute respiratory syndrome coronavirus 2; SFU, spot-forming unit; FITC, fluorescein isothiocyanate; DAPI, 4′,6-diamino-2-phenylindol; DMSO, dimethyl sulfoxide; VOC, variant of concern.
